# DSas-6 and Ana2 Coassemble into Tubules to Promote Centriole Duplication and Engagement

**DOI:** 10.1016/j.devcel.2010.11.010

**Published:** 2010-12-14

**Authors:** Naomi R. Stevens, Hélio Roque, Jordan W. Raff

**Affiliations:** 1 Sir William Dunn School of Pathology, University of Oxford, South Parks Road, Oxford OX1 3RE, UK

## Abstract

Centrioles form cilia and centrosomes, organelles whose dysfunction is increasingly linked to human disease. Centriole duplication relies on a few conserved proteins (ZYG-1/Sak/Plk4, SAS-6, SAS-5/Ana2, and SAS-4), and is often initiated by the formation of an inner “cartwheel” structure. Here, we show that overexpressed *Drosophila* Sas-6 and Ana2 coassemble into extended tubules (SAStubules) that bear a striking structural resemblance to the inner cartwheel of the centriole. SAStubules specifically interact with centriole proximal ends, but extra DSas-6/Ana2 is only recruited onto centrioles when Sak/Plk4 kinase is also overexpressed. This extra centriolar DSas-6/Ana2 induces centriole overduplication and, surprisingly, increased centriole cohesion. Intriguingly, we observe tubules that are structurally similar to SAStubules linking the engaged centrioles in normal wild-type cells. We conclude that DSas-6 and Ana2 normally cooperate to drive the formation of the centriole inner cartwheel and that they promote both centriole duplication and centriole cohesion in a Sak/Plk4-dependent manner.

## Highlights

► DSas-6 and Ana2 overexpression produces tubules resembling centriole cartwheels ► Sak promotes extra DSas-6/Ana2 loading onto centrioles, inducing overduplication ► Extra centriolar DSas-6/Ana2 also leads to increased centriole cohesion ► DSas-6/Ana2 tubules appear to regulate both centriole duplication and cohesion

## Introduction

Centrioles are microtubule (MT)-based cylinders required for the formation of two cellular structures: centrosomes and cilia. It is crucial that centriole numbers are tightly controlled. Centriole absence leads to catastrophic errors during early embryogenesis in both flies (Stevens et al., 2007) and worms (O'Connell et al., 2001), and centrosome and cilia dysfunction has been linked to a growing number of human diseases (Nigg and Raff, 2009). Moreover, excessive numbers of centrioles lead to tumorigenesis in flies (Basto et al., 2008) and have been linked to chromosomal instability in human cells (Ganem et al., 2009).

To ensure accurate regulation of centriole numbers, centriole duplication is closely coupled to the cell cycle. During mitosis centrosomes form the poles of the spindle, and each centrosome consists of a pair of “engaged” centrioles that are arranged at right angles to one another and are surrounded by an electron dense pericentriolar material (PCM). At the end of mitosis, the centrioles within a pair lose their strict orthogonal arrangement and move apart in a process known as “disengagement.” During S phase the disengaged centrioles duplicate, and a new, engaged, “daughter” centriole grows orthogonally out from the proximal end of each pre-existing “mother” centriole. It has been proposed that centriole disengagement at the end of mitosis acts as a “license” that allows centriole duplication in the following S phase (Loncarek et al., 2008, Tsou and Stearns, 2006a, Tsou and Stearns, 2006b).

Centriole duplication is best understood in worms, where a series of genome-wide RNA interference (RNAi) and genetic screens have identified just five proteins essential for this process—SPD-2, ZYG-1, SAS-5, SAS-6, and SAS-4 (Dammermann et al., 2004, Delattre et al., 2004, Kemp et al., 2004, Kirkham et al., 2003, Leidel et al., 2005, Leidel and Gonczy, 2003, O'Connell et al., 2001, Pelletier et al., 2004). SPD-2 is at the top of the hierarchy and is required to recruit the kinase ZYG-1 to the centriole (Delattre et al., 2006, Pelletier et al., 2006). Subsequently, a complex of SAS-5 and SAS-6 is recruited, and these proteins are required to form the large “central tube” that is the earliest observable intermediate in worm centriole formation (Pelletier et al., 2006). SAS-5 and SAS-6 in turn recruit SAS-4 (Dammermann et al., 2004, Delattre et al., 2004, Leidel et al., 2005), which allows the centriolar MTs to assemble around the central tube (Pelletier et al., 2006).

In flies there are also five proteins that appear to be most intimately involved in centriole duplication (Dobbelaere et al., 2008, Stevens et al., 2010). Four of these are related to the worm components—DSas-6 and DSas-4 are the *Drosophila* homologs of SAS-6 and SAS-4 (Basto et al., 2006, Peel et al., 2007, Rodrigues-Martins et al., 2007); whereas Ana2 and the kinase Sak/Plk4 are thought to be the functional orthologs of SAS-5 and ZYG-1, respectively (Bettencourt-Dias et al., 2005, Stevens et al., 2010). DSpd-2 is not required for centriole duplication in *Drosophila* (Dix and Raff, 2007, Giansanti et al., 2008), but flies have one additional duplication factor, Asterless (Asl) (Blachon et al., 2008, Stevens et al., 2010), which has not been identified in worms.

Although the factors required for centriole duplication are generally conserved between worms and other organisms (Nigg and Raff, 2009), there are significant differences in centriole structure. For example in many organisms (including flies and humans) a key early intermediate in centriole formation is the inner cartwheel, which consists of a central hub (that is smaller than the “central tube” observed in *C*. *elegans* centrioles) from which nine radial spokes emanate toward the centriolar MTs (Anderson and Brenner, 1971, Cavalier-Smith, 1974, Guichard et al., 2010). Therefore, it is unclear to what extent the detailed molecular pathway for centriole duplication elucidated in *C*. *elegans* applies to other organisms.

*Drosophila* spermatogenesis offers several advantages as a system for studying centriole duplication. Primary spermatocytes contain two large V-shaped pairs of engaged centrioles that are easily visible by light microscopy. In addition, unlike several other *Drosophila* cell types (Peel et al., 2007), the overexpression of any one of the five centriole duplication proteins leads to only very limited centriole overduplication (in the case of Ana2) or to no overduplication at all (in the case of Sak, DSas-6, Asl, or DSas-4). This suggests that several, or all, of the duplication proteins are present at near-limiting amounts in these cells. Therefore, we reasoned that overexpressing combinations of the duplication proteins might provide mechanistic insight into how they cooperate to drive centriole duplication.

## Results

### Overexpressing Individual Centriole Duplication Factors Does Not Perturb Centriole Behavior in Spermatocytes

To test the potential usefulness of overexpressing combinations of centriole duplication proteins in spermatocytes, we first examined the consequences of overexpressing each of the five duplication factors individually. Although a GFP-Ana2 transgene can drive limited centriole overduplication when two copies are present (Stevens et al., 2010), none of the duplication proteins detectably interfered with any aspect of centriole duplication or centriole behavior when just one copy of a transgene was present (see Figures S1A–S1E available online). As reported previously, all of the GFP-fusion proteins localized to centrioles (Basto et al., 2006, Dobbelaere et al., 2008, Goshima et al., 2007, Peel et al., 2007, Rodrigues-Martins et al., 2007). Although GFP-Sak and Asl-GFP were only detectable at centrioles (Figures S1A and S1E), cytoplasmic particles of Ana2-GFP, GFP-DSas-6, and DSas-4-GFP were also detectable (arrows in Figures S1B–S1D). None of these particles appeared to affect the endogenous centrioles.

### DSas-6 and Ana2 Coassemble to Form Highly Ordered Tubules that Resemble the Inner Cartwheel of the Centriole

**Figure 1:**
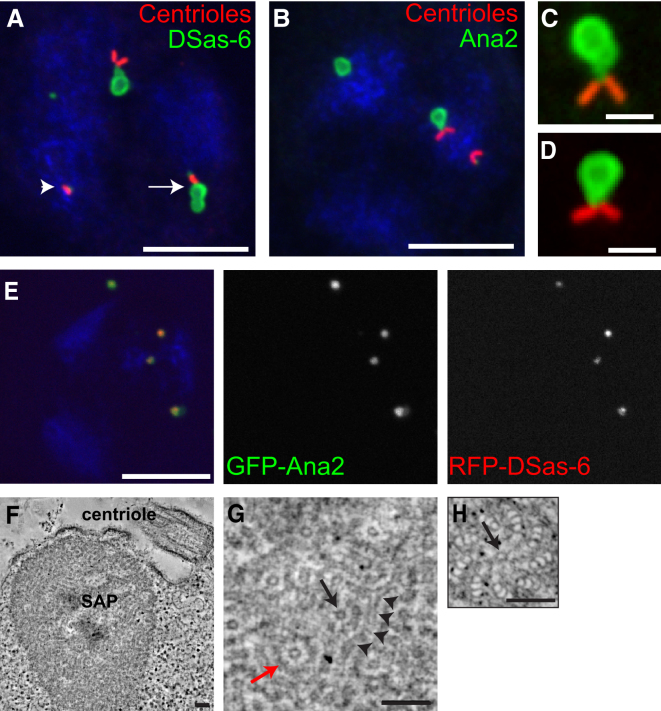
DSas-6 and Ana2 Coassemble into Highly Ordered Tubules that Resemble the Centriole Cartwheel (A–D) Mature primary spermatocytes expressing both GFP-DSas-6 and Ana2-GFP stained for the centriole marker GTU88^∗^ (red), DNA (blue), and DSas-6 (green in A and C) or Ana2 (green in B and D). DSas-6 and Ana2 antibodies reveal the presence of large particles that often appear to be inserting between the paired centrioles. The arrow and arrowhead highlight a prematurely disengaged centriole pair, where one centriole has remained associated with the SAP (arrow), whereas the other has not (arrowhead). (E) A mature primary spermatocyte expressing RFP-DSas-6 (red) and GFP-Ana2 (green) stained for DNA (blue). RFP-DSas-6 and GFP-Ana2 colocalize to the same cytoplasmic particles. (F and G) Images show low (F) and high (G) magnification images of a SAP taken from an electron tomogram (see Movie S1 for entire tomogram). The SAPs are highly structured and consist of a well-defined central tubule (a “SAStubule”; viewed in cross section, black arrow, or in transverse section, black arrowheads) linked by spokes to an electron-dense outer ring (red arrow). (H) An image from an electron tomogram showing a cross section through a spermatocyte centriole. Note the similarity between the central hub and spokes of the inner cartwheel (arrow in H) and the SAStubule and spokes of the SAP (black arrow in G). Scale bars, 10 μm (A), (B), and (E); 2 μm (C) and (D); and 100 nm (F)–(H). See also Figure S1 and Movies S1–S3.

We previously showed that Ana2 and DSas-6 can interact physically and functionally in other *Drosophila* tissues (Stevens et al., 2010). Therefore, we first investigated the consequences of overexpressing these two proteins together during spermatogenesis. Primary spermatocytes expressing one copy each of GFP-DSas-6 and Ana2-GFP contained a small number of large cytoplasmic particles (hereafter *S*as6/*A*na2 *p*articles [SAPs]) that stained with both Ana2 and DSas-6 antibodies (Figures 1A–1D), indicating that both proteins were coassembled into the same structures (this was confirmed in spermatocytes coexpressing RFP-DSas-6 and GFP-Ana2) (Figure 1E). Strikingly, the SAPs often associated with centrioles and, in particular, with the junction between the proximal ends of the two centrioles (Figures 1A–1D). We compared the localization of the SAPs with the cytoplasmic particles found in primary spermatocytes expressing two copies of GFP-DSas-6 alone. Both types of primary spermatocytes showed a similar number of cytoplasmic particles (an average of 2.21 SAPs per cell compared with 2.97 particles per GFP-DSas-6/GFP-DSas-6 cell), but in the GFP-DSas-6/GFP-DSas-6 cells, only ∼1% of particles (n = 101) touched a centriole, whereas ∼40% of SAPs (n = 75) were in contact with a centriole.

To better understand the nature of the SAPs, we examined sections of pupal testes by electron tomography (ET) (see Supplemental Experimental Procedures for all numbers of samples examined by electron microscopy [EM] and ET). In cells expressing GFP-DSas-6 and Ana2-GFP, we observed highly ordered cytoplasmic structures (Figure 1F) that were composed of a well-defined central tubule—viewed in cross section (black arrow, Figure 1G) or in transverse section (black arrowheads, Figure 1G)—linked by several spokes to an electron-dense outer ring (red arrow, Figure 1G) (see Movie S1 for full tomogram and Figures S1F–S1H for a schematic representation). These central tubules (hereafter “SAStubules” for *S*AS-6, *A*na2/*S*AS-5 tubules) appeared to wrap around themselves, sharing their outer rings with nearby SAStubules to form tightly packed particles.

Most strikingly, the SAStubules were very similar in shape and size to the hub of the centriolar cartwheel structure (arrow, Figure 1H) (average outer diameter of SAStubule = 13.35 nm, SD = ± 1.48 nm; average outer diameter of cartwheel central hub = 15.45 nm, SD = ± 1.23 nm), whereas the spokes radiating from the SAStubules closely resembled the spokes radiating from the central hub of the cartwheel (Figures 1G and 1H; although in both structures we usually failed to observe nine well-defined spokes). These observations strongly suggest that Ana2 and DSas-6 normally cooperate to drive the formation of the cartwheel during centriole duplication. In support of this conclusion, we failed to observe such well-organized tubules in the cytoplasmic particles formed when GFP-DSas-6 or Ana2-GFP were expressed individually in spermatocytes (Figures S1I and S1J and Movies S2 and S3).

### SAStubules Appear to Play a Role in Centriole Engagement

**Figure 2:**
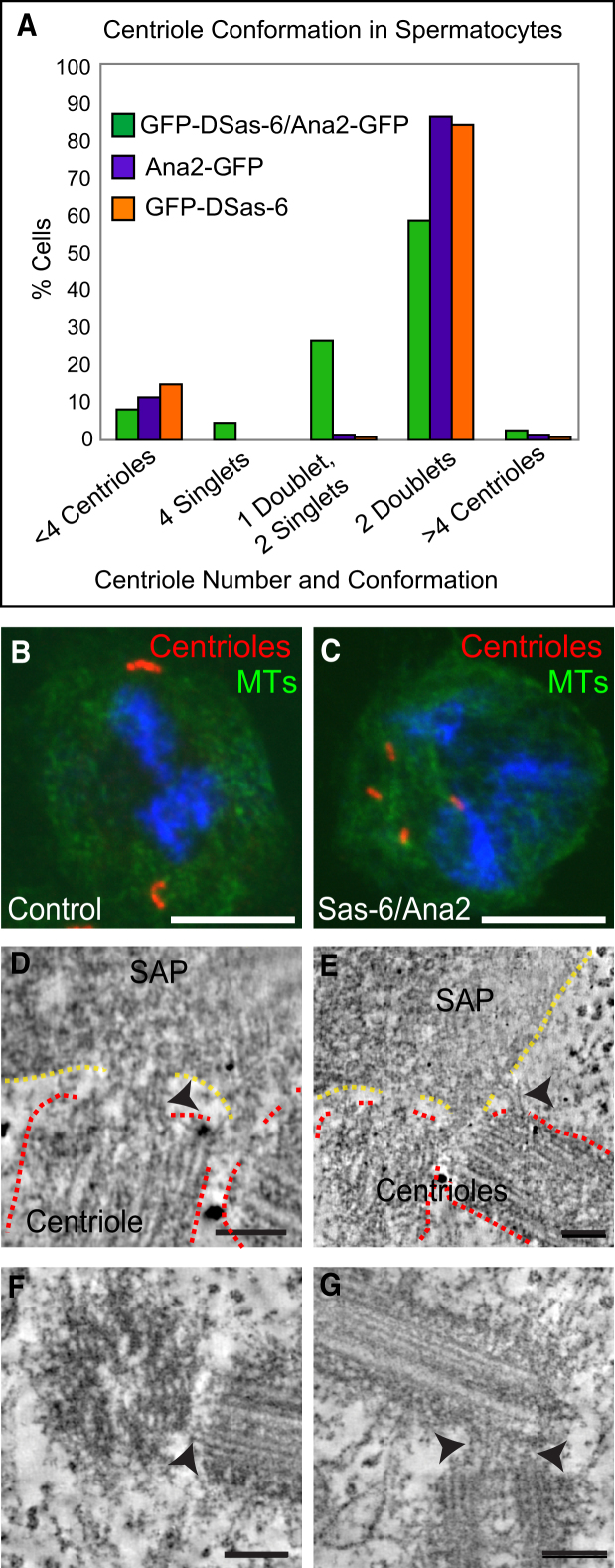
SAStubules Appear to Play a Role in Centriole Engagement (A) Quantification of centriole number and conformation in mature primary spermatocytes expressing either GFP-DSas-6 (n = 141), Ana2-GFP (n = 141), or both GFP-DSas-6 and Ana2-GFP (n = 197). In ∼30% of spermatocytes coexpressing GFP-DSas-6 and Ana2-GFP, one or both centriole pairs prematurely disengaged, giving rise to centriole singlets rather than the normal doublets. (B and C) Mature primary spermatocytes expressing either GFP-DSas-6 alone (B) or both GFP-DSas-6 and Ana2-GFP (C) stained for Asl (red), DNA (blue), and tubulin (green). In (C) the centrioles have prematurely separated to produce four singlets. (D and E) Images taken from an electron tomogram showing the interaction between a SAP (outlined by dotted yellow lines) and the proximal ends of an engaged centriole pair (outlined by dotted red lines) (see Movie S4 for entire tomogram). Note how SAStubules extend away from the SAP to the centrioles (arrowheads). (F and G) Images taken from electron tomograms that show the interaction between engaged centrioles in WT spermatocytes (see Movies S5 and S6 for entire tomograms). Note how tubules similar to the SAStubules link the engaged centrioles (arrowheads). Scale bars, 10 μm (B) and (C); and 100 nm (D)–(G). See also Movies S4–S6.

We noticed that the SAPs associated with the proximal ends of the centrioles often inserted themselves between the centrioles in a way that appeared to drive premature centriole disengagement: one or both of the centriole pairs had prematurely disengaged in ∼30% of these spermatocytes, with the SAPs usually remaining linked to one or both of the disengaged centrioles (arrow and arrowhead, Figures 1A and 2A–2C). We reasoned that the SAPs might interact with the centrioles in such a way that the SAP-centriole interaction displaced the normal centriole-centriole interaction, suggesting that these interactions are mechanistically similar. In support of this possibility, we found that although the SAPs were often linked to the proximal ends of centrioles in mature primary spermatocytes (∼40% of SAPs [n = 75] were in contact with a centriole in such cells), this association was usually lost as cells proceeded through meiosis I (∼6% of SAPs [n = 34] were in contact with a centriole in cells that had passed through meiosis I). Thus, the interaction between SAPs and centrioles appears to be lost at the same time that the centriole-centriole interaction is normally lost during centriole disengagement.

We used ET to further characterize the SAP-centriole interaction. We observed several instances where SAPs were interacting with endogenous centrioles, and in all cases multiple SAStubules extended away from the SAP to make direct contact with the centriole (Figures 2D and 2E; Movie S4). DSas-6 and Ana2 are normally concentrated in the proximal regions of the centrioles, and in *DSas-6* mutant spermatocytes, mother and daughter centrioles disengage prematurely (Rodrigues-Martins et al., 2007). Therefore, we wondered whether DSas-6 and Ana2 might normally form tubules that link engaged mother and daughter centrioles. Therefore, we examined electron tomograms of centrioles in wild-type (WT) spermatocytes. We consistently observed short tubules linking the centrioles, and these usually extended from the inner region of one of the centrioles (that appeared to be the daughter because its proximal end was closely abutted to the side of the other centriole) to the outer surface of the other centriole (presumably the mother) (Figures 2F and 2G; Movies S5 and S6).

Taken together, these data show that SAStubules form connections with centrioles that resemble the endogenous connections between engaged centrioles in both appearance and regulation. This supports the hypothesis that, at endogenous levels, DSas-6 and Ana2 coassemble into tubules that play an intimate role in centriole engagement.

### Extra Sak Can Recruit Extra DSas-6 and Ana2 onto Centrioles, Promoting Centriole Duplication and Centriole Cohesion

**Figure 3:**
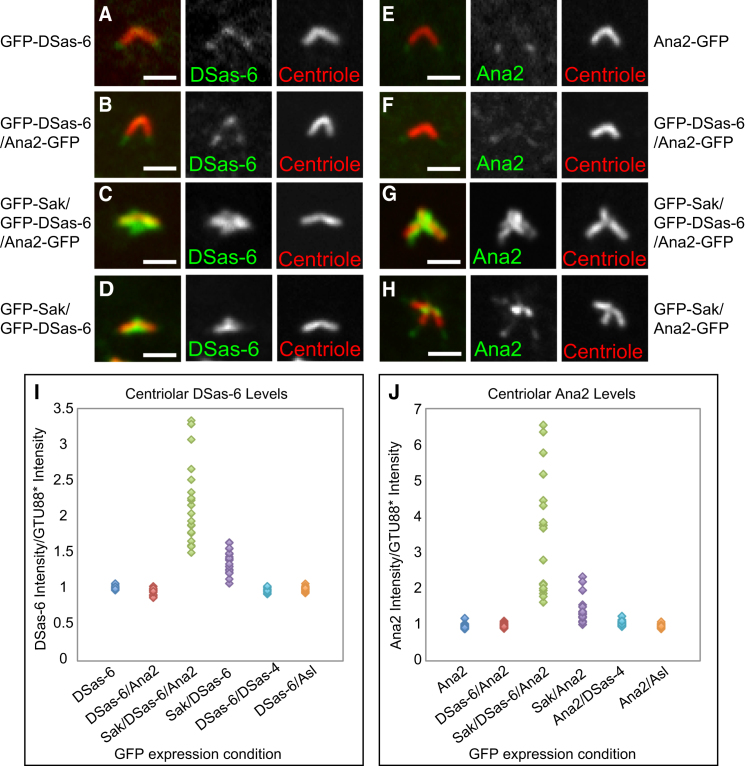
Extra Sak Recruits Extra DSas-6 and Ana2 onto Centrioles (A–H) Centrioles from mature primary spermatocytes expressing the GFP-fusion proteins indicated to the side of the panels and stained for GTU88^∗^ (red) and either DSas-6 (green in A–D) or Ana2 (green in E–H). (I and J) Graphs quantifying the amount of DSas-6 (I) or Ana2 (J) recruited to the centrioles when the various GFP-fusion proteins are expressed (as indicated on the x axis). A total of 20 centriole pairs from four testes were quantified for each condition. Note how the coexpression of GFP-DSas-6 and Ana2-GFP (B and F) does not detectably increase the amount of Ana2 or DSas-6 recruited to the centrioles over that of the single expression conditions (A and E). However, when GFP-Sak is also expressed, very large amounts of both Ana2 and DSas-6 are recruited to the centrioles (C and G). Sak can recruit extra DSas-6 (D) or Ana2 (H) to centrioles independently of overexpression of the other protein but not as strongly as when all three proteins are overexpressed together (C and G). The overexpression of DSas-4 or Asl with either DSas-6 or Ana2 did not appear to affect the centriolar recruitment of DSas-6 or Ana2 (I and J). Scale bars, 2 μm. See also Figure S2.

**Figure 4:**
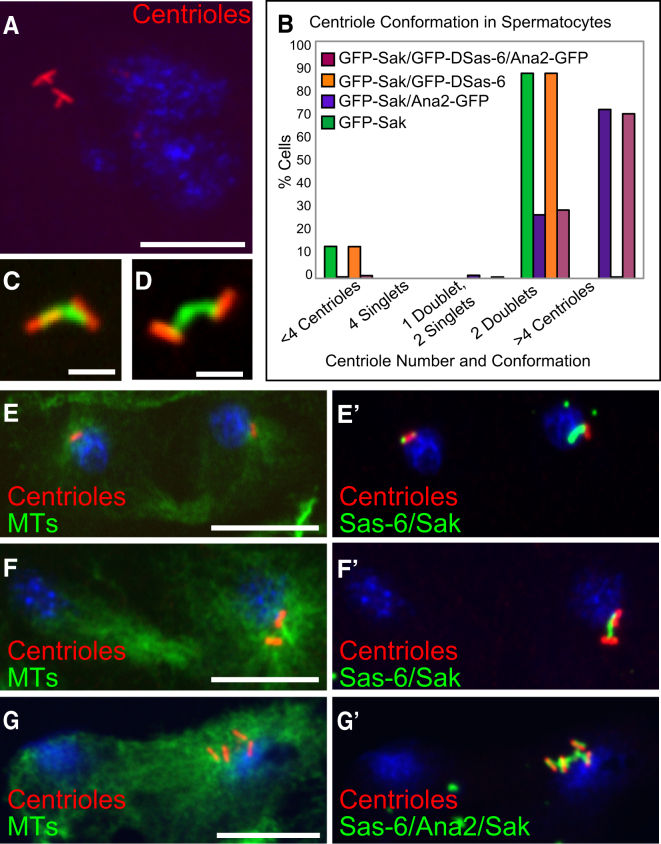
Extra Centriolar Ana2 and DSas-6 Promote Centriole Overduplication and Increased Centriole Cohesion (A) A mature primary spermatocyte expressing GFP-Sak, GFP-DSas-6, and Ana2-GFP stained for GTU88^∗^ (red) and DNA (blue). (B) Quantification of centriole number and conformation in mature primary spermatocytes expressing either GFP-Sak alone (n = 168), both GFP-Sak and Ana2-GFP (n = 153), both GFP-Sak and GFP-DSas-6 (n = 164), or GFP-Sak, Ana2-GFP, and GFP-DSas-6 (n = 170). (C) Centrioles from a spermatocyte in prophase of meiosis II expressing both GFP-DSas-6 and GFP-Sak and stained for the centriole marker Ana1 (red) with GFP shown in green. The centrioles are starting to disengage, but they appear to be held together by a GFP-containing linker. (D) Centrioles from a spermatocyte in telophase of meiosis II expressing both GFP-DSas-6 and GFP-Sak and stained for GTU88^∗^ (red) and DSas-6 (green). By this stage of meiosis, the linker has become extended into a fiber joining the mother and daughter centrioles. (E and F) Spermatocytes in telophase of meiosis II expressing both GFP-DSas-6 and GFP-Sak stained for Asl (red) and tubulin (green). In (E′) and (F′) GFP is shown in green. One centriole should be segregated into each daughter cell (E), but over half the time, two centrioles are mis-segregated into one daughter (F). This appears to be because the centrioles are joined by the fiber (F′), which is present in (E′) but has been severed allowing correct segregation. (G) Spermatocyte in telophase of meiosis II expressing GFP-Sak, Ana2-GFP, and GFP-DSas-6 stained for Ana1 (red), DNA (blue), and tubulin (green). In (G′) GFP is shown in green. The four centrioles are mis-segregating into one daughter cell due to being connected by GFP-containing fibers. Scale bars, 10 μm in (A) and (E)–(G) and 2 μm in (C) and (D).

Although the SAPs formed stable interactions with the proximal ends of centrioles, there was no increase in the amount of Ana2 or DSas-6 actually loaded onto the centrioles in the GFP-DSas-6/Ana2-GFP expressing cells relative to cells overexpressing either protein individually (Figures 3A, 3B, 3E, 3F, 3I, and 3J), and centrioles did not overduplicate (Figure 2A). In *C*. *elegans*, ZYG-1 acts to recruit SAS-5 and SAS-6 to centrioles (Delattre et al., 2006, Pelletier et al., 2006). Therefore, we wondered whether extra Sak might be required to induce loading of SAPs onto centrioles in a way that could drive centriole overduplication. To test this possibility, we generated flies carrying one copy each of GFP-Sak, GFP-DSas-6, and Ana2-GFP. Under these conditions, virtually all of the endogenous centrioles became coated in a large meshwork of Ana2-GFP and GFP-DSas-6, and ∼70% of cells appeared to have overduplicated their centrioles, forming clusters of centriole triplets or quadruplets (Figures 3C, 3G, 3I, 3J, 4A, and 4B). These “extra” centrioles appeared to be bona fide centrioles because they stained for multiple centriole markers (GTU88^∗^, Ana1, Ana2, and DSas-6; Figures 3G and 4A; data not shown), were of similar shape and size to the endogenous centrioles when stained with these markers, and recruited PCM and organized MT asters during meiosis (data not shown; Stevens et al., [2010]). Thus, extra Sak is required to load extra GFP-DSas-6 and Ana2-GFP onto centrioles in a way that can drive centriole overduplication.

Intriguingly, as the centrioles in these cells disengaged from one another at the end of meiosis I, the meshwork of GFP-DSas-6 and Ana2-GFP that covered the centrioles became stretched into long fibers that continued to link the disengaged centrioles even after the cells had passed through meiosis II, leading to extensive centriole mis-segregation (Figure 4G). Thus, extra Sak allows the SAPs to load onto centrioles in a way that not only promotes centriole overduplication but also promotes abnormal centriole cohesion.

To test whether extra Sak was capable of recruiting extra DSas-6 or Ana2 to centrioles independently of overexpression of the other, we examined spermatocytes expressing GFP-Sak together with either GFP-DSas-6 or Ana2-GFP. We observed that the levels of DSas-6 or Ana2 at the centrioles, particularly at the proximal region, were higher in these cells than in spermatocytes expressing either GFP-DSas-6 or Ana2-GFP alone (Figures 3D and 3H–3J) but were much lower than in cells expressing all three proteins together (Figures 3C, 3G, 3I, and 3J). Thus, extra Sak recruits extra Ana2 and extra DSas-6 to centrioles most strongly when they are overexpressed together.

Consistent with Ana2 levels acting to limit centriole overduplication in primary spermatocytes (Stevens et al., 2010), the extra Ana2-GFP recruited to the centrioles in GFP-Sak/Ana2-GFP cells appeared to induce the formation of extra centrioles (assessed by the criteria described above) (Figure 4B), presumably because there is sufficient excess endogenous DSas-6 in these cells to allow limited centriole overduplication. In support of this interpretation, the percentage of cells with centriole quadruplets (as opposed to triplets) was lower in the GFP-Sak/Ana2-GFP cells (∼1%, n = 153) compared to GFP-Sak/GFP-DSas-6/Ana2-GFP cells (∼8%, n = 170), indicating that Ana2 is more efficient at inducing centriole overduplication when DSas-6 is also overexpressed. Intriguingly, in ∼58% (n = 93) of cells expressing GFP-Sak and GFP-DSas-6, the centrioles remained linked together by a DSas-6 and Ana2 containing fiber throughout meiosis II (Figures 4C–4F), in a manner similar to that seen in GFP-Sak/GFP-DSas-6/Ana2-GFP cells (Figure 4G). This suggests that extra Sak can recruit extra DSas-6 to centrioles in a way that promotes abnormal centriole cohesion without stimulating centriole overduplication (presumably because Ana2 remains limiting for duplication).

### Overexpressing DSas-4 or Asl with Sak, DSas-6, or Ana2 Does Not Perturb Centriole Behavior in Spermatocytes

The results described above indicate that Sak, Ana2, and DSas-6 cooperate to drive the earliest events of centriole duplication. Therefore, we wondered where DSas-4 and Asl fit into the pathway. The combined expression of DSas-4-GFP or Asl-GFP with either GFP-Sak, GFP-DSas-6, Ana2-GFP, or with each other had no detectable effect on the centrioles in primary spermatocytes. Neither protein was able to recruit observable amounts of extra Ana2 or DSas-6 to centrioles (Figures 3I and 3J), and no centriole overduplication or centriole linker formation was observed in any of these combinations (Figure S2; data not shown). These results suggest that DSas-4 and Asl may not be intimately involved in initiating cartwheel formation.

## Discussion

Here, we have overexpressed combinations of the five *Drosophila* centriole duplication proteins to gain insight into how these proteins function together. Our results provide important clues as to how three of these proteins, Sak, DSas-6, and Ana2, cooperate to drive the early events of centriole duplication and also reveal an unexpected potential link between centriole duplication and centriole engagement.

The earliest steps of centriole duplication are best understood in *C*. *elegans*, where the kinase ZYG-1 recruits SAS-6 and SAS-5 to form a large “central tube”; SAS-4 is subsequently required to add MTs around the central tube to form a new daughter centriole (Pelletier et al., 2006). However, centrioles in most other species lack a central tube and, instead, contain a more complex “cartwheel” structure composed of a small central hub that radiates nine spoke-like projections toward the outer arrays of centriolar MTs (Anderson and Brenner, 1971, Cavalier-Smith, 1974, Guichard et al., 2010). Although the pathway of centriole assembly is not well defined in other species, the cartwheel appears to be an essential early intermediate, and SAS-6 homologs are required for its formation (Culver et al., 2009, Kilburn et al., 2007, Nakazawa et al., 2007). Although DSas-6 can oligomerize in vitro (Gopalakrishnan et al., 2010) and assemble into higher order structures when overexpressed in vivo (Peel et al., 2007, Rodrigues-Martins et al., 2007), the relationship between these DSas-6 structures and centrioles is unclear. Here, we have shown that, when co-overexpressed with Ana2, DSas-6 can assemble into highly ordered structures that bear a striking resemblance to the inner cartwheel.

Our data strongly indicate that these structures are not simply an artifact of overexpression because the SAStubules interact specifically with the proximal ends of the endogenous centrioles, often inserting themselves between mother and daughter centrioles and forcing their premature disengagement. Most strikingly, if Sak is also overexpressed with DSas-6 and Ana2, these proteins are loaded onto the centrioles and can drive centriole overduplication. These findings suggest that, despite their morphological diversity, centrioles in worms and flies initiate centriole duplication in the same way, with Sak/ZYG-1 stimulating the assembly of a SAS-6 and Ana2/SAS-5 based tubule structure (the central tube in worms, and the more elaborate cartwheel in flies) at the proximal end of the mother centriole.

Unexpectedly, our data also suggest that these key duplication proteins are also involved in maintaining the cohesion between engaged mother and daughter centrioles. Our ET analysis of engaged centrioles in WT spermatocytes has revealed the existence of short tubules that appear to link engaged mother and daughter centrioles. These tubules resemble the SAStubules that we observe linking the SAPs to the centrioles, although it is difficult to get clear images of these structures in the closely spaced environment between the centrioles. Nevertheless, it is striking that the SAPs can force the premature disengagement of the endogenous centrioles, strongly suggesting that the SAP-centriole linkage simply displaces the endogenous centriole-centriole linkage.

In support of this conclusion, the connection between the SAPs and the centrioles is lost at the end of meiosis I, just as the endogenous centrioles normally disengage, indicating that the SAP-centriole interaction may be regulated by the same cell cycle cues that regulate the centriole-centriole interaction. Moreover, when Sak is co-overexpressed with DSas-6 and Ana2, centrioles overduplicate, but there is also an increase in centriole cohesion, with many of the duplicated centrioles disengaging from one another but ultimately failing to separate properly because they remain linked by DSas-6 and Ana2 containing fibers. Although the biological significance of these fibers remains to be determined, it is tempting to speculate that they represent an aberrant stabilization of the tubules that normally link engaged centrioles, particularly because DSas-6 is known to be required to maintain centriole engagement (Rodrigues-Martins et al., 2007).

Taken together, our observations support a role for DSas-6 and Ana2 in promoting both centriole duplication and centriole cohesion, with Sak acting as a crucial regulator of both processes. Cryo-ET on purified human centrosomes has revealed that these centrioles appear to be linked by a single “stalk” connecting the cartwheel central hub of the daughter centriole to the side of the mother (Guichard et al., 2010). Thus, in the simplest model, one SAStubule could act both to connect the engaged centrioles and as the hub for the centriole inner cartwheel (although our observations indicate that several tubules may normally link engaged mother and daughter centrioles in spermatocytes).

In summary our results suggest a mechanism for the earliest events of centriole duplication, giving important insight into the assembly of the crucial cartwheel intermediate. The next step will be to determine at the molecular level how Sak/Plk4 regulates the assembly of DSas-6 and Ana2 to drive the formation of such a precisely ordered structure.

## Experimental Procedures

### Fly Stocks

GFP-DSas-6, GFP-Sak, and DSas-4-GFP (Peel et al., 2007), Asl-GFP, Ana2-GFP, and GFP-Ana2 (Stevens et al., 2010), and RFP-DSas-6 (this work) transgenic lines all contain GFP or RFP fusions expressed from the Ubq promoter, which drives moderate expression in all tissues (Lee et al., 1988). Because we cannot detect the endogenous proteins due to their low levels, we cannot determine the precise level of overexpression in each case (Stevens et al., 2010).

### Antibodies for Immunofluorescence

The following antibodies were used: rabbit anti-DSas-6, 1:250 (Peel et al., 2007); rabbit anti-DSas-4, 1:250 (Basto et al., 2006); rabbit anti-Asl, 1:500 (Stevens et al., 2009); rabbit anti-Ana1, 1:500 (Stevens et al., 2009); rabbit anti-Ana2, 1:250 (Stevens et al., 2010); mouse monoclonal anti-α-tubulin, 1:1000 (DM1α; Sigma); and mouse GTU88^∗^, a batch of the GTU88 anti-γ-tubulin antibody (Sigma) that cross-reacts with centrioles in *Drosophila*, 1:1000 (Martinez-Campos et al., 2004). Alexa 488, Cy3, and Cy5 secondaries were from Molecular Probes or Jackson Laboratories.

### Fixed Analysis of Adult Testes

Testes from adult flies within 3 days of eclosion were dissected, fixed, and stained as described previously (Dix and Raff, 2007). Samples were observed on a PerkinElmer ERS Spinning Disc confocal system, mounted on an inverted microscope (Axiovert 200M; Carl Zeiss MicroImaging Inc.) with a charge-coupled device camera (Orca ER; Hamamatsu), using a 63×/1.25 NA objective. Images were acquired using Ultraview ERS software (PerkinElmer), imported into Photoshop CS2 (Adobe), and adjusted to use the full range of pixel intensities.

### ET

Late pupal testes were dissected in phosphate buffer and fixed in 2.5% glutaraldehyde in 0.1 M cacodylate buffer (pH 7.2) for 2 hr at 4°C. Testes were washed in buffer and post-fixed in 1% OsO_4_ followed by extensive washing in distilled water. Samples were then en-block stained with 0.5% uranyl acetate overnight at 4°C followed by washing in distilled water. Samples were dehydrated in an ethanol series and embedded in Agar100. Polymerization was at 60°C for 42 hr. Semi-thick serial sections (150 nm) were obtained in a Reichert-Jung Ultracut E (Leica Microsystems, Austria) and stained in lead citrate. For ET, colloidal gold particles of 15 nm were applied to both sides of the grid sections. Dual-axis tilt series (55°–55°) of the same area in adjacent sections were acquired in a TECNAI T12 transmission microscope (FEI, Netherlands) at 13,000× magnification with SerialEM (Mastronarde, 2005). Images were aligned and tomograms reconstructed by R-weighted back-projection with the user interface eTomo, and analysis was performed with the software package IMOD (Kremer et al., 1996, Mastronarde, 1997).

## Supplemental Information

Document S1. Two Figures and Supplemental Experimental Procedures

Movie S1. (Related to Figure 1). Overexpressed DSas-6 and Ana2 Coassemble into Particles Made Up of Highly Ordered Tubules This movie shows an electron tomogram of a SAP located close to the cortex of a mature primary spermatocyte expressing GFP-DSas-6 and Ana2-GFP. One of the centrioles in the cell is also visible in this tomogram (top right). The black dots visible at the surface of the tomogram are the gold particles spread on to the semi-thick section to allow alignment of the images. The SAStubules are linked by spokes to an electron dense outer ring and form a complex 3D meshwork that can best be visualised by playing the movie backwards and forwards while trying to follow the path of the individual SAStubules.

Movie S2. (Related to Figure 1 and S1). Particles Resulting from Overexpression of DSas-6 Alone Do Not Have a Well-Defined Structure This movie shows an electron tomogram of a cytoplasmic particle from a mature primary spermatocyte expressing only GFP-DSas-6. These particles lacked any well-ordered structure, making them difficult to identify in normal preparations. We therefore used immuno-EM with an anti-GFP antibody to unambiguously identify these particles. In this movie, tomograms from three serial sections have been joined together so that nearly the entire volume of the particle is contained within the tomogram. The gold particles used in the immuno-EM to identify the structures (and also to align the images) can be visualised at the surface of each new section. Note how this particle appears to be hollow in the middle, and several areas within the particle are largely devoid of any electron dense material.

Movie S3. (Related to Figure 1 and S1). Particles Resulting from Overexpression of Ana2 Alone Do Not Have a Well-Defined Structure This movie shows an electron tomogram of a cytoplasmic particle from a mature primary spermatocyte expressing only Ana2-GFP. These particles lacked any well-ordered structure, making them difficult to identify in normal preparations. We therefore used immuno-EM with an anti-GFP antibody to unambiguously identify these particles. In this movie, tomograms from five serial sections have been joined together so that nearly the entire volume of the particle is contained within the tomogram. The gold particles used in the immuno-EM to identify the structures (and also to align the images) can be visualised at the surface of each new section.

Movie S4. (Related to Figure 2). SAStubules Extend Away from SAPs to Contact Centrioles This movie shows an electron tomogram of a SAP from a mature primary spermatocyte expressing GFP-DSas-6 and Ana2-GFP. The SAP is directly contacting the proximal ends of an engaged centriole pair. In this movie, tomograms from two serial sections have been joined together. The black dots visible at the surface of each tomogram are the gold particles spread on to the semi-thick section to allow alignment of the images. By playing the movie backwards and forwards one can visualise an extensive meshwork of SAStubules that radiate away from the SAP and appear to make intimate contact with both the inner and outer surface of the engaged centrioles.

Movie S5. (Related to Figure 2). Tubules Similar to SAStubules Appear to Link Engaged Centrioles in Wild-Type Cells These movies show electron tomograms of engaged centriole pairs in WT mature primary spermatocytes. Although it is difficult to resolve structure within the small gap between the proximal ends of the centrioles, by playing the movies backwards and forwards one can identify a small number of tubule structures that appear to link the centrioles.

Movie S6. (Related to Figure 2). Tubules Similar to SAStubules Appear to Link Engaged Centrioles in Wild-Type Cells These movies show electron tomograms of engaged centriole pairs in WT mature primary spermatocytes. Although it is difficult to resolve structure within the small gap between the proximal ends of the centrioles, by playing the movies backwards and forwards one can identify a small number of tubule structures that appear to link the centrioles.
