# Supplementary material for: DSas-6 and Ana2 Coassemble into Tubules to Promote Centriole Duplication and Engagement
Source: Dev Cell. 2010 Dec 14;19(6):913–9. doi: 10.1016/j.devcel.2010.11.010 (PMC4159445; doi:10.1016/j.devcel.2010.11.010)
Supplement: Document S1. Two Figures and Supplemental Experimental Procedures [file mmc1.pdf]

Developmental Cell *19*

Supplemental Information

DSas-6 and Ana2 Coassemble into Tubules to Promote Centriole Duplication and Engagement

Naomi R. Stevens, Hélio Roque, and Jordan W. Raff

FIGURE S1

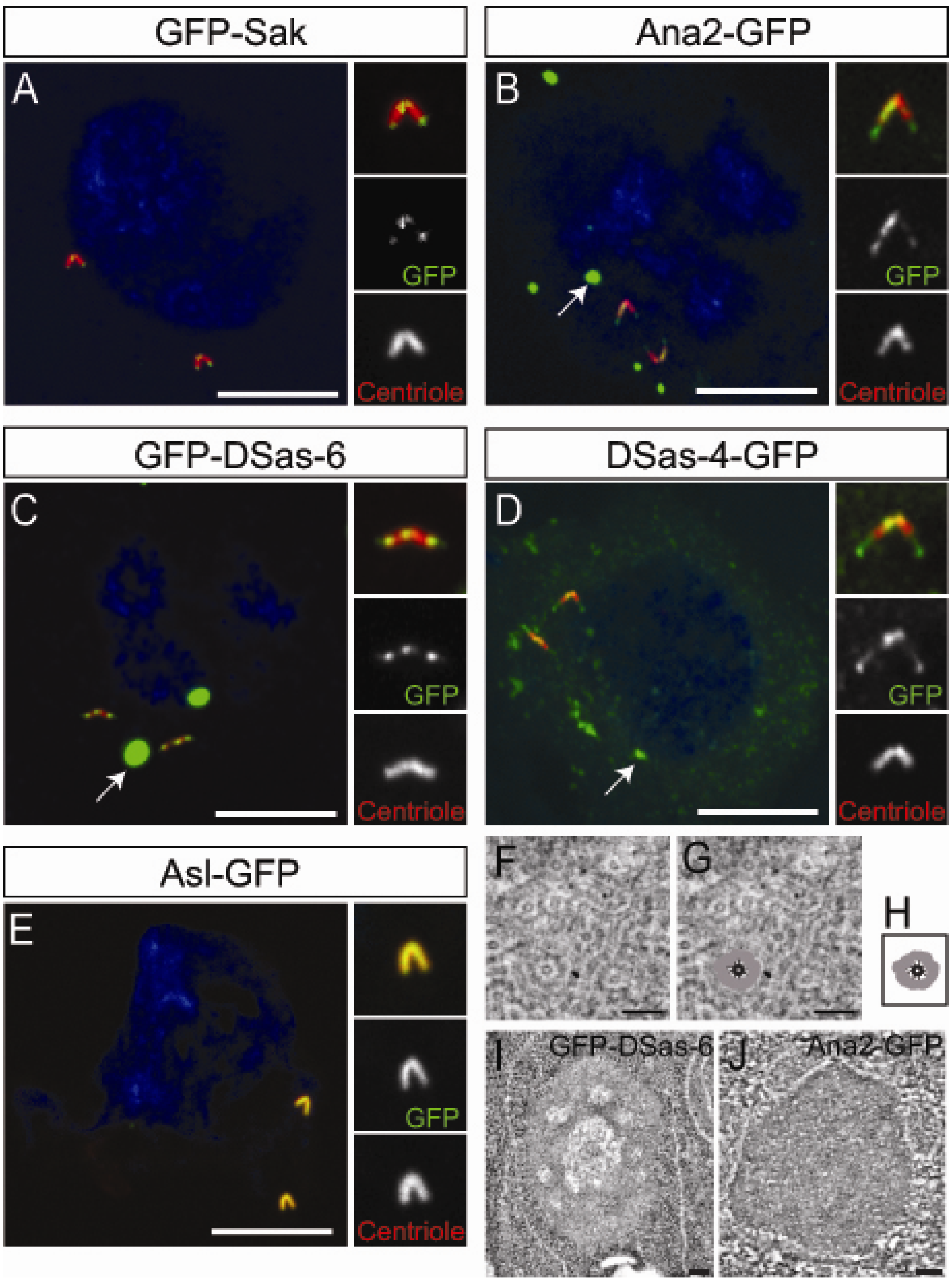

Figure S1 (Related to Figure 1): Analysis of the particles formed by the overexpression of individual centriole duplication proteins compared to the combined expression of DSas-6 and Ana2

(A-E) Mature primary spermatocytes expressing either GFP-Sak (A), Ana2-GFP (B), GFP-DSas-6 (C), DSas-4-GFP (D) or Asl-GFP (E) and stained for

the centriole marker GTU88* (red) and DNA (blue); GFP is in green. Magnified images of one centriole pair are shown alongside. No centriolar phenotypes are observed when any of the five transgenes is expressed alone as a single copy. Note the presence of cytoplasmic particles (arrows) containing the GFP-protein fusions in (B-D). (F) An image from an electron tomogram showing a high magnification view of a SAP (as shown in Figure 1G); (G) overlayed on this image is a schematic representation of the SAStubule (central black circle) that is similar to the “hub” of the centriole cartwheel. Nine spokes (black lines) emanate from the SAStubule, although, in practice, it is difficult to discern all 9 spokes, and this is also true of the hub in the real centriole cartwheel (see Figure 1H). The electron-dense “outer ring” that surrounds the SAStubule is shown in grey. (H) The schematic overlay shown on its own. (I,J) Images taken from electron tomograms showing the cytoplasmic particles formed by the expression of GFP-DSas-6 (I) or Ana2-GFP (J) in mature primary spermatocytes. These particles lacked the highly ordered structure of the SAPs, so we used immuno-electron microscopy (EM) with an anti-GFP antibody to unambiguously identify these particles. Although the gold particles used to identify these structures are not visible in the images shown here, they are visible in the full tomograms (see Movies S2 and S3). Scale bars (A-E) = 10 µm (F,G, I, J) = 100nm.

FIGURE S2

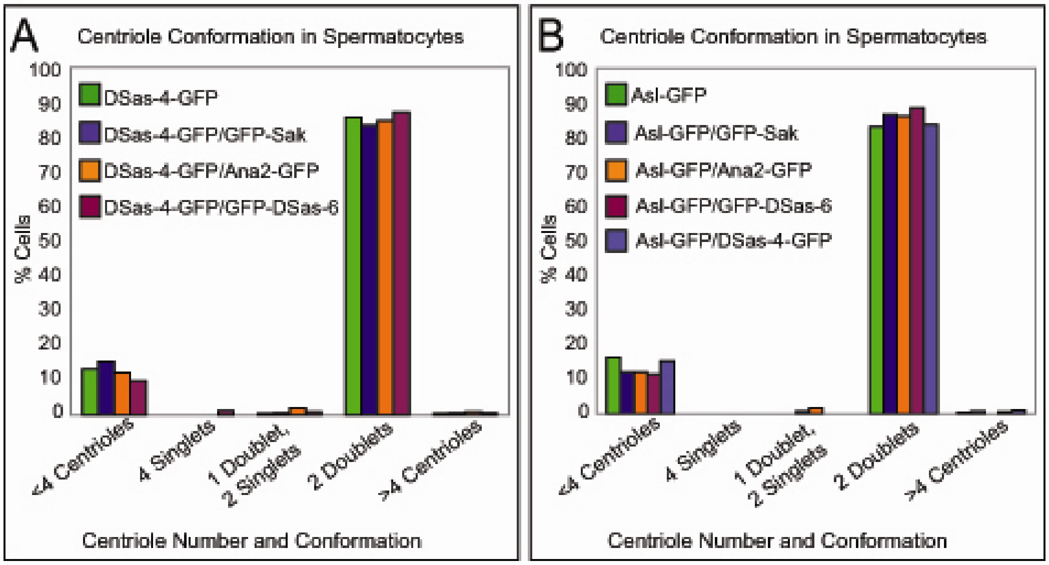

Figure S2 (Related to Figure 3): Overexpressing DSas-4 or Asl with Sak, DSas-6 or Ana2 does not perturb centriole behaviour in spermatocytes

(A) Quantification of centriole number and conformation in mature primary spermatocytes expressing either DSas-4-GFP alone (n=195), or DSas-4-GFP with either GFP-Sak (n=162), Ana2-GFP (n=189) or GFP-DSas-6 (n=294). No obvious centriolar phenotype is observed under any of the conditions. (B) Quantification of centriole number and conformation in mature primary

spermatocytes expressing either Asl-GFP alone (n=190), or Asl-GFP with either GFP-Sak (n=142), Ana2-GFP (n=232), GFP-DSas-6 (n=174) or DSas-4-GFP (n=191). No obvious centriolar phenotype is observed under any of the conditions.

SUPPLEMENTAL EXPERIMENTAL PROCEDURES

Quantification of GFP-DSas-6 particles and SAPs in primary spermatocytes

Cytoplasmic particles were quantified in fixed samples of primary spermatocytes expressing either one copy of Ubq-Ana2-GFP and one copy of Ubq-GFP-DSas-6 or two copies of Ubq-GFP-DSas-6. Stacks of images spanning the entire cell volume were taken at 0.2 μm intervals using the confocal system described above. Cytoplasmic particles were identified semi-automatically using Volocity software (Improvision) in projections of these stacks. The number of particles per cell and whether the particle touched a centriole were recorded. A total of 34 cells were analysed taken from 4 testes for each condition.

Quantification of centriolar levels of Ana2 and DSas-6

Centriolar levels of Ana2 were quantified in fixed samples of mature primary spermatocytes expressing either Ana2-GFP, GFP-Sak/Ana2-GFP, GFP-DSas-6/Ana2-GFP, GFP-Sak/GFP-DSas-6/Ana2-GFP, Ana2-GFP/DSas-4-GFP or Ana2-GFP/Asl-GFP and centriolar levels of DSas-6 were quantified in primary spermatocytes expressing either GFP-DSas-6, GFP-DSas-6/Ana2-GFP, GFP-Sak/GFP-DSas-6, GFP-Sak/GFP-DSas-6/Ana2-GFP, GFP-DSas-6/DSas-4-GFP or GFP-DSas-6/Asl-GFP. Stacks of images spanning the centrioles were taken as described above. Centrioles (defined by GTU88* staining) were identified using Volocity software (Improvision) in projections of these stacks. The total fluorescence intensity of the Ana2 or DSas-6 staining overlapping with the GTU88* staining was measured for each of 20 centriole pairs from four testes per condition and divided by the GTU88* intensity. Note that this method is likely to underestimate the levels of extra DSas-6 and extra Ana2 recruited to centrioles particularly in the triple GFP-Sak/GFP-DSas-6/Ana2-GFP expressing spermatocytes as in these cells the DSas-6 and Ana2 staining extends well beyond the GTU88* staining.

Numbers of structures analysed by EM and ET

The following numbers of structures were analysed first by EM and then by ET. Centriole pairs in WT cells: ~20 analysed by EM, 5 analysed by ET; SAPs: >20 analysed by EM (of which ~10 were associated with centrioles), 5 analysed by ET (of which 3 were associated with centrioles); GFP-DSas-6 particles: 3 analysed by immuno-EM, 1 analysed by ET; Ana2-GFP particles: 5 analysed by immuno-EM, 1 analysed by ET.

Immuno-electron tomography

For immuno-labelling of Ana2-GFP or GFP-DSas-6 particles, testes were fixed in 4% paraformaldehyde in 0.1M calcodylate buffer (pH 7.2) for 2h at

4˚C. Samples were dehydrated in an ethanol series at 4˚C and embedded in LRWhite resin at 37˚C over 7 days. Semi-thick sections were obtained as above. Grids were floated in a drop of PBS with 1% BSA and 20mM glycine for 15 min followed by blocking in PBS and 1% BSA for 10 min. Next the grids were floated in a drop of 1:300 dilution of rabbit anti-GFP antibody (a kind gift of John Kilmartin) in blocking solution for 1hr at RT. Grids were washed in PBS for 15 mins and floated in a drop of 1:50 dilution of goat anti-rabbit IgG conjugated with 10 nm gold (BBInternational, UK) for 1hr at RT. Grids were washed in PBS for 30 min and rinsed in distilled water for 5 min, and tomograms were obtained as described above. As a control, WT testes were treated in the same way, and this confirmed that no immuno-gold-labelled particles could be found in WT testes.
